# Biomedical data analyses facilitated by open cheminformatics workflows

**DOI:** 10.1186/s13321-023-00718-8

**Published:** 2023-04-17

**Authors:** Eva Nittinger, Alex Clark, Anna Gaulton, Barbara Zdrazil

**Affiliations:** 1 Medicinal Chemistry, Research and Early Development, Respiratory and Immunology (R&I), BioPharmaceuticals R&D, AstraZeneca, Gothenburg, Sweden; 2 Research Informatics, Collaborative Drug Discovery, Inc., Ottawa, Canada; 3 Data Operations, Exscientia, Oxford, UK; 4 European Molecular Biology Laboratory, European Bioinformatics Institute (EMBL-EBI), Hinxton, UK

The Special Collection ”Biomedical Data Analyses Facilitated by Open Cheminformatics Workflows” (https://www.biomedcentral.com/collections/BDAOCW) aimed to collect and publish cheminformatics workflows for curation and analysis of diverse life science data sets. Especially at a time where in many areas of science reproducibility of results is significantly challenged [1, 2], it is important to encourage publication of workflows for data curation, including data extraction, integration, annotation, cleaning/filtering, standardization and analysis. However, this reproducibility ”crisis” is not only a challenge but can become an opportunity for change and better publication practise in the future [3].

For many scientific workflows, curation of data is essential and takes a significant amount of time. Many different scientific disciplines and data types depend on data standardization and preprocessing, which is nicely exemplified by the different areas covered in this special issue - from small molecules, to metabolomics, and drug-protein interactions. However, choices made during data curation can be quite subjective, i.e. containing user-defined cut-offs, and also depends on the problem at hand. Thus, published workflows shall enable comparability and reproducibility of results in line with FAIR (findable, accessible, interoperable, and reusable) principles both for data [4] as well as software [5]. Another advantage of using already existing workflows is avoiding mistakes and challenges others have already faced and overcome before.

Many scientific studies in the fields of cheminformatics and computational chemistry aim to extract and connect knowledge from (experimental) data. One fundamental assumption is the correctness of input data from experimental resources. However, systematic errors, i.e. translation between 1D, 2D, and 3D structure representations, as well as random errors, such as incorrect human input, occur ranging from on average two errors per (medicinal chemistry) publication to 0.1$-$3.4% for different databases [6–8]. In addition to errors in experimental resources, the correct representation and standardization of molecules, including their tautomers and protonation states, can be highly challenging and time-consuming. Molecules are often represented by the Simplified Molecular Input Line Entry System (SMILES) [9] or InChI [10, 11] representation. However, there is no universal standard for SMILES and using different programs will lead to different representations for the same molecule. The importance of (automated) chemical structure curation is demonstrated by the publication of structure standardization workflows by major bioactivity data resources like ChEMBL [12], PubChem [13], or canSAR [14].

In the field of machine learning (ML) and artificial intelligence (AI) publication of code is more commonly applied. Due to increasing amount of published methods in that area, more publications including guidelines on reproducibility but also on model comparison itself became available [15–17]. It could serve as an example for other areas of cheminformatics and computational chemistry for which open source publications and workflows are not yet commonly published with manuscripts.

Publication of data on the other hand is an even more challenging topic especially when considering proprietary data. Public bioacitivty data often have a lower number of negative or inactive data compared to proprietary data, thus displaying a higher ratio of actives to inactives than commonly seen in i.e. high-throughput screening (HTS) runs [18–21]. Thus, public and proprietary data sets complement each other in terms of chemical space coverage. Another advantage of proprietary data is the estimation of experimental uncertainty, since often a more homogeneous curation pipeline and assay setup is applied as well as multiple measurements for the same compounds are available. In order to satisfy the request for reproducibility and data sharing without violating intellectual property (IP) rights, the application of developed methods and workflows to public and private data in the same manner is a good solution. This process has been encouraged for research papers submitted to J. Cheminf. as demonstrated by these examples [22, 23].

The workflows submitted for this special issue include KNIME workflows [24], Galaxy [25] or Jupyter notebooks[26]. In addition to these workflow tools, platforms for publishing and sharing of code, such as GitHub [27] or GitLab are available and allow sharing with and enhancements by peers. Larger data that requires more storage space, such as model input data or machine learning models themselves, can be stored in open-repositories such as Zenodo [28]. Docker [29] became popular in order to avoid any issues with cross platform installation. With all these resources available, ideal conditions for improvements and requirements for reproducibility are at hand.

Ultimately, publication of workflows does not mean that these workflows cannot be changed anymore. These serve as a basis and starting point for further research and can also help during teaching, with already existing initiatives such as TeachOpenCADD [30, 31]. Thus, we strongly encourage open access publication of workflows in order to help driving research the best way possible.

In this special issue, diverse topics were covered from data analysis (nonadditivity analysis, thermal shift assay analysis, or MS/MS analysis for metabolomics), structural analysis (drug-protein interactions, fragment-based virtual screening) to machine learning (retraining of ML models, ML for off-target predictions, MMPA and QSAR). This nicely illustrates how important data workflows and analysis are across different scientfic fields.

As mentioned already, data availability is still a great challenge especially when it comes to high quality data. Many of todays’ influential researchers have grown up in a culture where data and knowledge sharing has not been appreciated yet, but was rather seen as potentially limiting their chances for securing one of the rare tenured academic positions. Herein, a reward system to encourage data sharing could be a first incentive. Additionally, data sharing initiatives, such as federated learning with the MELLODDY project [32], have been conducted to share proprietary data and enhance machine learning models across companies. In the future, it would be great to see more initiatives to share data cross company but also between academia and industry to advance method development.

## Abbreviations

AI: Artificial intelligence
CADD: Computer-aided drug design
FAIR: Findable, accessible, interoperable, and reusable
HTS: High-throughput screening
InChI: International chemical identifier
IP: Intellectual property
ML: Machine learning
MMPA: Matched molecular pair analysis
SMILES: Simplified molecular input line entry system
QSAR: Quantitative structure activity relationship

## References

[1] Yang Y, Youyou W, Uzzi B (2020). Estimating the deep replicability of scientific findings using human and artificial intelligence. Proc Natl Acad Sci.

[2] Errington TM, Denis A, Perfito N, Iorns E, Nosek BA (2021). Challenges for assessing replicability in preclinical cancer biology. eLife.

[3] Munafò M, Chambers C, Collins A, Fortunato L, Macleod M (2022). The reproducibility debate is an opportunity, not a crisis. BMC Res Notes.

[4] Wilkinson MD, Dumontier M, Aalbersberg IJ, Appleton G, Axton M, Baak A, Blomberg N, Boiten J-W, da Silva Santos LB, Bourne PE, Bouwman J, Brookes AJ, Clark T, Crosas M, Dillo I, Dumon O, Edmunds S, Evelo CT, Finkers R, Gonzalez-Beltran A, Gray AJG, Groth P, Goble C, Grethe JS, Heringa J, ’t Hoen PAC, Hooft R, Kuhn T, Kok R, Kok J, Lusher SJ, Martone ME, Mons A, Packer AL, Persson B, Rocca-Serra P, Roos M, van Schaik R, Sansone S-A, Schultes E, Sengstag T, Slater T, Strawn G, Swertz MA, Thompson M, van der Lei J, van Mulligen E, Velterop J, Waagmeester A, Wittenburg P, Wolstencroft K, Zhao J, Mons B (2016). The FAIR guiding principles for scientific data management and stewardship. Sci Data.

[5] Barker M, Chue Hong NP, Katz DS et al (2022) Introducing the FAIR principles for research software. Sci Data 9: 622. 10.1038/s41597-022-01710-x 10.1038/s41597-022-01710-x PMC9562067 36241754

[6] Olah M, Mracec M, Ostopovici L, Rad R, Bora A, Hadaruga N, Olah I, Banda M, Simon Z, Mracec M, Oprea TI (2005). Wombat: world of molecular bioactivity. Chemoinform Drug Discov.

[7] Young D, Martin T, Venkatapathy R, Harten P (2008). Are the chemical structures in your qsar correct?. QSAR Comb Sci.

[8] Fourches D, Muratov E, Tropsha A (2010). Trust, but verify: on the importance of chemical structure curation in cheminformatics and qsar modeling research. J Chem Inform Model.

[9] Weininger D (2002). Smiles, a chemical language and information system. 1. introduction to methodology and encoding rules. J Chem Inform Comput Sci.

[10] Heller S, McNaught A, Stein S, Tchekhovskoi D, Pletnev I (2013). Inchi - the worldwide chemical structure identifier standard. J Cheminform.

[11] Heller SR, McNaught A, Pletnev I, Stein S, Tchekhovskoi D (2015). Inchi, the iupac international chemical identifier. J Cheminform.

[12] Bento AP, Hersey A, Félix E, Landrum G, Gaulton A, Atkinson F, Bellis LJ, Veij MD, Leach AR (2020). An open source chemical structure curation pipeline using rdkit. J Cheminform.

[13] Hähnke VD, Kim S, Bolton EE (2018). Pubchem chemical structure standardization. J Cheminform.

[14] Dolciami D, Villasclaras-Fernandez E, Kannas C, Meniconi M, Al-Lazikani B, Antolin AA (2022). Cansar chemistry registration and standardization pipeline. J Cheminform.

[15] Walters WP (2020). Code sharing in the open science era. J Chem Inf Model.

[16] Bajorath J, Coley CW, Landon MR, Walters WP, Zheng M (2021). Reproducibility, reusability, and community efforts in artificial intelligence research. Artif Intel Life Sci.

[17] Walters WP (2022). Comparing classification models-a practical tutorial. J Comput Aided Mol Des.

[18] Bradley D (2008). Dealing with a data dilemma. Nature Rev Drug Discov.

[19] Rodríguez-Pérez R, Miyao T, Jasial S, Vogt M, Bajorath J (2018). Prediction of compound profiling matrices using machine learning. ACS Omega.

[20] Cáceres EL, Mew NC, Keiser MJ (2020). Adding stochastic negative examples into machine learning improves molecular bioactivity prediction. J Chem Inf Model.

[21] Valsecchi C, Grisoni F, Motta S, Bonati L, Ballabio D (2020). NURA: a curated dataset of nuclear receptor modulators. Tox Appl Pharmaco.

[22] Morger A, Mathea M, Achenbach JH, Wolf A, Buesen R, Schleifer K-J, Landsiedel R, Volkamer A (2020). KnowTox: pipeline and case study for confident prediction of potential toxic effects of compounds in early phases of development. J Cheminf.

[23] Boldini D, Friedrich L, Kuhn D, Sieber SA (2022). Tuning gradient boosting for imbalanced bioassay modelling with custom loss functions. J Cheminform.

[24] Berthold MR, Cebron N, Dill F, Gabriel TR, Kötter T, Meinl T, Ohl P, Thiel K, Wiswedel B (2009). Knime - the konstanz information miner: Version 20 and beyond. SIGKDD Explor Newsl.

[25] Afgan E, Baker D, Batut B, van den Beek M, Bouvier D, Čech M, Chilton J, Clements D, Coraor N, Grüning BA, Guerler A, Hillman-Jackson J, Hiltemann S, Jalili V, Rasche H, Soranzo N, Goecks J, Taylor J, Nekrutenko A, Blankenberg D (2018). The Galaxy platform for accessible, reproducible and collaborative biomedical analyses: 2018 update. Nucleic Acids Res.

[26] Kluyver T, Ragan-Kelley B, Pérez F, Granger B, Bussonnier M, Frederic J, Kelley K, Hamrick J, Grout J, Corlay S, Ivanov P, Avila D, Abdalla S, Willing C, Loizides F, Scmidt B, Development team J (2016). Jupyter notebooks - a publishing format for reproducible computational workflows. Positioning and power in academic publishing: players, agents and agendas.

[27] github (2023). GitHub. Retrieved from https://github.com/

[28] European Organization For Nuclear Research, OpenAIRE (2013). Zenodo. CERN.

[29] Merkel D (2014). Docker: lightweight linux containers for consistent development and deployment. Linux J.

[30] Sydow D, Rodríguez-Guerra J, Volkamer A (2021). Teaching computer-aided drug design using TeachOpenCADD.

[31] Sydow D, Rodríguez-Guerra J, Kimber TB, Schaller D, Taylor CJ, Chen Y, Leja M, Misra S, Wichmann M, Ariamajd A, Volkamer A (2022). TeachOpenCADD 2022: open source and FAIR Python pipelines to assist in structural bioinformatics and cheminformatics research. Nucleic Acids Res.

[32] Oldenhof M, Ács G, Pejo B, Schuffenhauer A, Holway N, Sturm N, Dieckmann A, Fortmeier O, Boniface E, Mayer C, Gohier A, Schmidtke P, Niwayama R, Kopecky D, Mervin L, Rathi PC, Friedrich L, Formanek A, Antal P, Rahaman J, Zalewski A, Heyndrickx W, Oluoch E, Stössel M, Vanco M, Endico D, Gelus F, de Boisfossé T, Darbier A, Nicollet A, Blottière M, Telenczuk M, Nguyen VT, Martinez T, Boillet C, Moutet K, Picosson A, Gasser A, Djafar I, Simon A, Arany A, Simm J, Moreau Y, Engkvist O, Ceulemans H, Marini C, Galtier M (2022). Industry-scale orchestrated federated learning for drug discovery. arXiv.

